# Disruption of phosphatidyl choline metabolism affects gametophyte development in *Arabidopsis thaliana*

**DOI:** 10.1093/plphys/kiag232

**Published:** 2026-04-21

**Authors:** Mary Williams, Mamoona Khan

**Affiliations:** American Society of Plant Biologists; Assistant Features Editor, Plant Physiology, American Society of Plant Biologists; Department of Plant Pathology, Institute of Crop Science and Resource Conservation (INRES), University of Bonn, Nussallee 9, Bonn 53115, Germany

Phospholipids are essential structural and functional components of plant cell membranes. They maintain membrane integrity, enable cellular compartmentalization, and play key roles in signaling processes (Nakamura 2017). Phosphatidylcholine (PC) is one of the most abundant phospholipids and is continuously remodeled through lipid turnover processes. One major route involves deacylation of PC, which results in the production of glycerophosphocholine (GPC). GPC is subsequently hydrolyzed by glycerophosphodiester phosphodiesterases (GDPDs), generating glycerol-3-phosphate (G3P) and free choline ((Fig. 1a; Ohshima et al. 2008). G3P serves as a central metabolic intermediate linking lipid turnover to primary metabolism and de novo lipid biosynthesis, while choline can be reused for PC resynthesis, contributing to lipid homeostasis (Hejazian et al. 2024).

**Figure 1:**
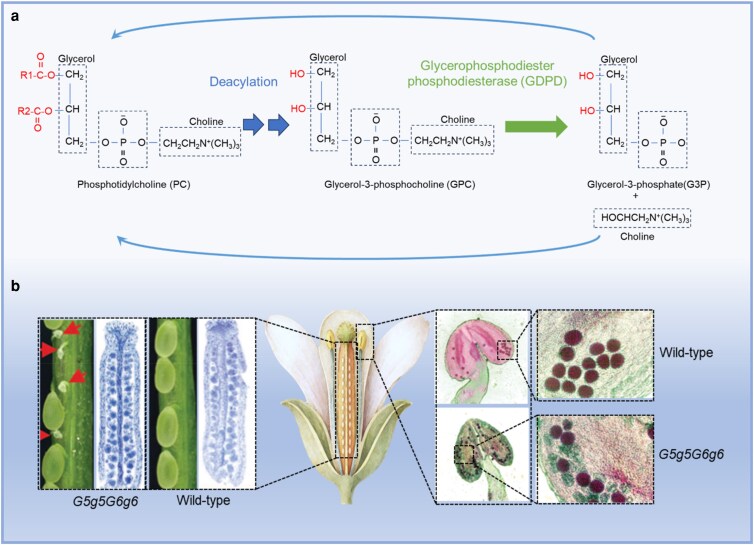
Glycerophosphodiester phosphodiesterases GDPD5 and GDPD6 regulate phospholipid homeostasis and gametophyte development in *Arabidopsis thaliana*. a) Schematic representation of phosphatidylcholine (PC) turnover and recycling in plant cells. PC serves as a central node for acyl editing. A fraction of PC undergoes degradation to glycerophosphocholine (GPC), which is hydrolyzed by GDPD5 and GDPD6 to produce glycerol-3-phosphate (G3P) and choline. These metabolites are subsequently recycled for PC resynthesis. Simplified from Siebers et al. (2026) Fig. 9. b) Representative phenotypes of gametophytes and siliques of *gdpd5x gdpd6* heterozygous double mutants (*G5g5G6g6*) compared to wild-type plants, showing defective pollen development and seed abortion. Phenotypic pictures are taken from Figs. 4 and 5 of Siebers et al. (2026).

Although GDPDs are evolutionarily conserved across bacteria, fungi, animals, and plants (Corda et al. 2014), their physiological roles and regulatory mechanisms in plants remain comparatively less understood. In *Arabidopsis thaliana*, several GDPD family members are transcriptionally induced under phosphate deficiency, supporting their involvement in adaptive nutrient remobilization and lipid remodeling (Mehra and Giri 2016).

In the recent issue of *Plant Physiology*, Siebers et al. (2026) examined the roles of 2 so far unstudied *A. thaliana* GDPDs in phospholipid turnover during phosphate deprivation. Analysis of single and double mutants showed no major involvement in overall phospholipid turnover under normal or phosphate-deficient conditions. Instead, the study identified a specific role in maintaining PC homeostasis in anthers, with both enzymes being essential for proper male and female gametophyte development.

Siebers et al. (2026) first assessed the biochemical activity of GDPD5 and GDPD6 by transiently expressing both proteins in *Nicotiana benthamiana* and performing GDPD assays using microsomal fractions. Both enzymes showed clear activity, with a preference for GPC, indicating functional GDPD activity. To determine their subcellular localization, GDPD5- and GDPD6-eGFP fusion proteins were analyzed by confocal microscopy. Both proteins displayed a fluorescence pattern that co-localized with an endoplasmic reticulum (ER) marker, demonstrating ER association.

The authors next obtained multiple *A. thaliana* insertion mutants for *GDPD5* and *GDPD6* to assess their in vivo function. Two independent alleles were identified for each gene. Homozygous single mutants displayed no visible growth or developmental phenotypes. RT-PCR analyses revealed that *gdpd5-1* and *gdpd6-1* represent null alleles, whereas *gdpd5-2* and *gdpd6-2* retain partial expression of truncated transcripts and residual protein activity. To explore the metabolic impact of GDPD5 and GDPD6, the authors then quantified G3P levels and profiled membrane lipids under phosphate-limited conditions. Loss of either enzyme led to a noticeable reduction in G3P, supporting their contribution to glycerophosphodiester breakdown in vivo (Fig. 1a). However, despite this decrease, the overall lipid composition remained largely unchanged in the mutants compared to wild type under both sufficient and limiting phosphate supply. This suggests that, although GDPD5 and GDPD6 contribute to G3P production, their activity is not a major determinant of bulk membrane lipid remodeling during phosphate stress.

To test whether GDPD5 and GDPD6 have overlapping roles, the authors crossed the single mutants to generate double mutants. Segregation from the double heterozygote resulted in no double mutant progeny. Interestingly, it also revealed a suppression in the double wild-type progeny. The authors interpreted these data as indicating that during meiosis, the presence of a cell carrying both mutations rendered all spores inviable. These results demonstrate the importance of GDPD5 and GDPD6 during both sporogenesis and gametogenesis (Fig. 1b). Importantly, introducing a functional *GDPD6* gene restored fertility, confirming that the defect is caused by the combined loss of GDPD5 and GDPD6.

To further investigate reproductive defects, Siebers et al. (2026) examined seed and pollen development in double heterozygous plants. While early floral structures appeared normal under bright-field light microscopy, nearly half of the seeds failed to develop. This result indicated defects that became apparent only after fertilization. Further microscopic analyses revealed that anthers were slightly smaller and that about half of the pollen was nonviable (Fig. 1b). Functional assays demonstrated that these abnormal pollen grains failed to germinate.

Given the strong expression of *GDPD5* and *GDPD6* in reproductive tissues, the authors focused their lipid analysis on anthers. Using mass spectrometry, they found that total membrane lipid levels were reduced in mutant anthers, partly reflecting their smaller size. However, when looking at lipid composition, PC levels were significantly decreased, while most other membrane lipids remained largely unchanged. Minor shifts were observed in other lipids, but these were not as pronounced.

In contrast, storage lipids such as triacylglycerol (TAG), which are important for pollen germination, were not substantially affected in overall abundance, although slight changes in fatty acid composition were detected. Together, these results indicate that loss of *GDPD5* and *GDPD6* specifically disrupts PC homeostasis in anthers, rather than causing broad changes in lipid metabolism.

Altogether, these findings identify GDPD5 and GDPD6 as redundant enzymes that have a critical role during *A. thaliana* reproduction. They support gametophyte development by maintaining PC homeostasis in anthers rather than bulk lipid turnover. Future studies should examine how altered PC or choline metabolism affects membrane formation during meiosis and gametogenesis using cell-type-specific lipid and metabolite analyses.

## Recent related articles in *Plant Physiology*

- Shomo et al. (2024) reviewed the impact of low temperatures on plant survival, highlighting disruptions to cellular processes and adaptive changes in membrane lipid composition under cold stress.
- Song et al. (2024) demonstrated that pollen germination and tube growth depend on rapid phospholipid synthesis and remodeling mediated by 2 key enzymes and showed that pistil-derived lipids can support pollen tube growth and fertilization.
- Laxalt et al. (2025) reviewed phosphoinositide-specific phospholipase C (PI-PLC) signaling in plants.

## Data Availability

No data were generated or analyzed in this study.
